# Memory‐Based Deception Detection: Extending the Cognitive Signature of Lying From Instructed to Self‐Initiated Cheating

**DOI:** 10.1111/tops.12353

**Published:** 2018-06-15

**Authors:** Linda M. Geven, Gershon Ben‐Shakhar, Merel Kindt, Bruno Verschuere

**Affiliations:** ^1^ Department of Clinical Psychology University of Amsterdam; ^2^ Department of Psychology Hebrew University of Jerusalem

**Keywords:** Concealed Information Test (CIT), Memory detection, Deception, External validity, Cheating, Honesty

## Abstract

From a cognitive perspective, lying can be regarded as a complex cognitive process requiring the interplay of several executive functions. Meta‐analytic research on 114 studies encompassing 3,307 participants (Suchotzki, Verschuere, Van Bockstaele, Ben‐Shakhar, & Crombez, 2017) suggests that computerized paradigms can reliably assess the cognitive burden of lying, with large reaction time differences between lying and truth telling. These studies, however, lack a key ingredient of real‐life deception, namely self‐initiated behavior. Research participants have typically been instructed to commit a mock crime and conceal critical information, whereas in real life, people freely choose whether or not to engage in antisocial behavior. In this study, participants (*n *=* *433) engaged in a trivia quiz and were provided with a monetary incentive for high accuracy performance. Participants were randomly allocated to either a condition where they were instructed to cheat on the quiz (mimicking the typical laboratory set‐up) or to a condition in which they were provided with the opportunity to cheat, yet without explicit instructions to do so. Assessments of their response times in a subsequent Concealed Information Test (CIT) revealed that both instructed cheaters (*n *=* *107) and self‐initiated cheaters (*n *=* *142) showed the expected RT‐slowing for concealed information. The data indicate that the cognitive signature of lying is not restricted to explicitly instructed cheating, but it can also be observed for self‐initiated cheating. These findings are highly encouraging from an ecological validity perspective.

## Abstract 2

Geven, Ben‐Shakhar, Kindt and Verschuere point out that research on deception detection usually employs instructed cheating. They experimentally demonstrate that participants show slower reaction times for concealed information than for other information, regardless of whether they are explicitly instructed to cheat or whether they can freely choose to cheat or not. Finding this ‘cognitive signature of lying’ with self‐initiated cheating too is argued by the authors to strengthen the external validity of deception detection research. [75]

## 1 Introduction

### 1.1 A cognitive view on deception

Cognition‐based lie detection focuses on the assumption that lying is cognitively more demanding than truth telling. Liars often require a significant amount of mental processes in order to actively suppress the pre‐potent truth and appear innocent (Spence et al., 2001; Verschuere, Spruyt, Meijer, & Otgaar, 2011). People report to experience lying as associated with a larger cognitive effort than truth telling (Caso, Gnisci, Vrij, & Mann, 2005; Vrij, Semin, & Bull, 1996). A study tracking participants’ arm movements while moving a videogame remote to either a deceptive or truthful option on the screen, confirmed that lying takes longer than telling the truth (Duran, Dale, & McNamara, 2010; Spence et al., 2001; Walczyk, Roper, Seemann, & Humphrey, 2003). Neurocognitive research found that inhibiting the truth and constructing a lie are reflected by increased activation in several regions of the brain associated with cognitive control (e.g., iFG; see Abe, Suzuki, Mori, Itoh, & Fujii, 2007; Ganis, Kosslyn, Stose, Thompson, & Yurgelun‐Todd, 2003; Nunez, Casey, Egner, Hare, & Hirsch, 2005; Sip, Roepstorff, McGregor, & Frith, 2008). These findings inspired a new focus of lie detection techniques that aim to improve differentiation between liars and truth tellers by adding additional cognitive load, such as asking interviewees to tell their story in reversed order (Evans, Michael, Meissner, & Brandon, 2013; Vrij et al., 2008) or by maintaining eye contact (Vrij, Mann, Leal, & Fisher, 2010).

### 1.2 Response time as an index of cognitive effort involved in lying

Renewed attention has drawn researchers to the easily applicable behavioral measure of response time as an indicator for (increased inhibition associated with) deception. Although initial research, often using suboptimal measurement conditions, did not find strong effects of reaction times (RTs) as a deception measure (see Luria, 1932; Marston, 1920), the increased use of computerized measures led to revived research interest.

Seymour, Seifert, Shafto, and Mosmann (2000), for instance, found that RTs provide a fast and reliable indication of recognition of concealed information. Research participants committed a mock crime in the laboratory and were subsequently asked to do a seemingly unrelated binary classification task on the computer. While measuring RTs, participants had to indicate whether they recognized the stimuli presented in the Concealed Information Test (CIT; Lykken, 1959) by pressing one of two response keys. Critical details from the committed crime were intermixed with irrelevant, neutral items. Upon measuring response latencies on these critical details (*probes*) in comparison to irrelevant words, 89% of the guilty participants could be correctly classified as such.

Another study using a mock‐theft scenario, instructed participants to either steal a CD with exam questions or to read a newspaper article about the incident. In a subsequent autobiographical Implicit Association Task (aIAT; Sartori, Agosta, Zogmaister, Ferrara, & Castiello, 2008), participants had to classify sentences describing possible autobiographical events (e.g., ‘I am in front of a computer’ or ‘I am currently on the beach’) to either the ‘true’ or ‘false’ label by pressing one of two response keys. All 15 guilty participants made a faster association between the crime‐related questions (e.g., ‘I stole the CD with the exam questions’) and the label ‘true’, while 13 of the 15 innocent participants were faster combining denials of theft with ‘true’, leading to a ROC area of 0.96.

Meta‐analytic research on 114 studies encompassing 3,307 participants (Suchotzki et al., 2017) using various computerized RT paradigms, shows the potential of RTs to index deception, with a large effect size (Cohen's *d *=* *1.05). Yet the results are nearly exclusively based on non‐forensic samples and studies lacking key ingredients of real‐life deception.

### 1.3 On the limitations of instructed lying and cheating

Deception is commonly defined as a voluntary act (see Vrij, 2004), in which deliberation and intention are key factors. Moreover, Sip et al. (2008) pointed out that deception comes without forewarning and instructions on when to deceive. Yet, in laboratory studies on detecting deception, participants are often explicitly instructed to lie (e.g., Furedy, Davis, & Gurevich, 1988) or commit a staged crime and subsequently conceal knowledge (e.g., Lykken, 1959; Nahari & Ben‐Shakhar, 2011). As a consequence, questions can be raised about the suitability of these studies, involving explicitly instructed rule‐breaking, to mimic deception outside of the laboratory.

Cognitive and neuroscientific researchers have begun to explore the role of voluntary dishonest behavior (Blakemore, Winston, & Frith, 2004; Kozel et al., 2005; Mohamed et al., 2006; Sip et al., 2008). More recently, the field of *detecting* deception has also started to focus on more ecologically valid paradigms to mimic deceit and investigate its role in test sensitivity. For instance, Nahari, Breska, Elber, klein Selle, and Ben‐Shakhar (2017) let participants ‘choose’ to either enact a mock crime or an innocent computerized task. The study revealed a similar CIT detection efficiency, based on psychophysiological and RT measures, for those who choose to commit the mock crime as for participants who were explicitly ordered to commit the mock crime.

Yet in forced‐choice‐paradigms, even ‘deceptive’ participants are not guilty rule‐breakers, but in fact obediently complying with the experimenters’ instructions. It is pointed out that deception, in all its complexity, can only be fully investigated when the decision to deceive is based on the participants’ own initiative. The current study attempts to address this limitation by investigating whether the act of deliberate versus obedient cheating in this new paradigm influences the sensitivity of deception detection techniques based on RT measures.

### 1.4 Current study

The vast majority of deception studies have relied upon instructed deception. In an attempt to enhance external validity while maintaining experimental control, self‐initiated deception in the current study was defined as cheating on a trivia quiz. Self‐initiated cheaters were compared with instructed cheaters and non‐cheaters (i.e., fair players).

By borrowing successful cheating paradigms from the field of social psychology and behavioral economics (see DeAndrea, Carpenter, Shulman, & Levine, 2009; Domnich et al., 2015; Halevy, Shalvi, & Verschuere, 2014; Nagin & Pogarsky, 2003), participants in the current experiment were provided with a monetary incentive for high accuracy performance on a trivia quiz. Unbeknownst to the participants, the quiz was constructed in a way that it would be almost impossible to earn the bonus without looking up the correct answers on the last two questions.

Participants were randomly allocated to either a condition where they were instructed to cheat on the quiz (mimicking the typical laboratory set‐up) or to a condition in which they were provided with the opportunity to cheat, yet without explicit instructions to do so. In this manner, deceptive behavior was completely self‐initiated and involved the crucial deliberate intent to deceive. Moreover, contrary to most laboratory research, participants in the current study were not aware of the fact that they would partake in a memory detection test during the experiment. This way, critical items were incidentally acquired and thereby both encoding and retention resembled realistic settings (see Meixner & Rosenfeld, 2014).

By mirroring the conditions that would be present in a real‐world environment, individual differences in the decision to cheat were examined as a secondary aim of this study. According to Lee and Ashton (2012), individual differences in honesty and morality are a core component of personality, which they introduced as an additional factor to the Big Five personality inventory. This specific new factor, called Honesty‐Humility (HH) in the HEXACO Personality Inventory (HEXACO‐PI‐R; Lee & Ashton, 2004), taps into differences in the willingness to manipulate others for personal gain, the temptation to break rules and interest in social status. Across six studies, a consistent negative correlation was found between HH scores and the likelihood of cheating (Hilbig & Zettler, 2015). In the current study, HH scores of self‐initiated cheaters and fair players are compared as to further explore differences between participants who willingly cheat or not. It is expected that self‐initiated cheaters report to be more inclined to break rules for financial gain compared to fair players, reflected by lower HH scores in the HEXACO‐PI‐R. Furthermore, these two groups are compared to the instructed cheaters, who form the baseline condition for this particular measure. This allows us to clarify whether individual differences in cheating behavior are driven by personality attributes of the cheaters (as often assumed) or of the fair players.

## 2 Method

The study was approved by the ethical committee of the Department of Psychology of the University of Amsterdam (2016‐CP‐7217). The tasks scripts and data are available on https://osf.io/t9y7n.

### 2.1 Participants

The sample consisted of 433 students (79.4% female) from the University of Amsterdam. Their average age was 20.41 years old (*SD* age* *=* *2.41, range from 17 to 44). Participants were recruited through a university portal and received course credits as compensation. Participants were mainly students pursuing a degree in psychology (52%), communication science (21%) or psychobiology (21%). All participants provided consent before taking part in the study.

Participants were randomly allocated to the instructed cheating versus the possibility to cheat condition with a 1:3 ratio, as the latter condition was subsequently split in self‐initiated cheaters versus fair players.

One of the two cheating‐evoking questions of the trivia quiz asked about the author of the novel *Wishful Drinking*. However, halfway during data collection the author passed away, which evoked multiple news items occasionally mentioning her novel. Because of possible familiarity with the probe, we excluded the data of 44 participants that were tested in the week after Carrie Fisher's illness and death. For all subsequent participants, this trivia question was replaced with an equally difficult question.

#### 2.1.1 Instructed cheaters, self‐initiated cheaters, and fair players

Based upon the instructions and their performance on the cheating‐evoking trivia quiz questions, participants were labeled instructed cheaters (i.e., those given instructions to cheat), self‐initiated cheaters (i.e., those given opportunity to cheat and answered the two cheating‐evoking questions correctly) or fair players (i.e., those given opportunity to cheat and answered neither of the two cheating‐evoking questions correctly). Twenty‐two individuals answered only one of the two cheating‐evoking questions correctly and were excluded from further analysis due to an uncertain ground truth criterion regarding their cheating behavior.

There were 130 participants in the instructed cheating condition (17.7% male, *M* age* *=* *20.57, *SD* age* *=* *2.83), who completed the HEXACO and trivia quiz. CIT data were missing from three participants and 20 participants were excluded due to low target accuracy^1^ (i.e., an error rate of 50% or more on target items, see Kleinberg & Verschuere, 2015). The final sample for CIT analysis consisted therefore of 107 instructed cheaters (20.6% male, *M* age* *=* *20.72, *SD* age* *=* *3.03).

There were 259 participants in the possibility to cheat condition (22.8% male, *M* age* *=* *20.37, *SD* age* *=* *1.95). One hundred seventy‐five participants answered both cheating‐evoking questions of the trivia quiz correctly and were named the self‐initiated cheaters (23.4% male; *M* age* *=* *20.04, *SD* age* *=* *1.73). All completed the HEXACO and trivia quiz. CIT data were missing from eight participants and 25 participants were excluded due to low target accuracy. The final sample for CIT analysis consisted therefore of 142 self‐initiated cheaters (22.5% male, *M* age* *=* *19.94, *SD* age* *=* *1.65).

Sixty‐two participants (21% male; *M* age* *=* *20.59, *SD* age* *=* *2.32) did not know the correct answers to the difficult quiz‐questions and were therefore labeled fair players. One participant responded incorrectly to two out of three validation questions in the personality measure and was therefore excluded from HEXACO analysis. CIT data were missing from four participants and 13 participants were excluded due to low target accuracy. The final sample for CIT analysis therefore consisted of 45 fair players (17.8% male, *M* age* *=* *20.55, *SD* age* *=* *1.89).

There were no significant differences in gender between the three groups, *X* ^2^(2)* *=* *1.48, *p *=* *.477, φ_c_ = 0.06. Of all 237 participants in the possibility to cheat condition (partial cheaters excluded), 41 out of 54 male participants cheated on the trivia quiz (75.9%). For female participants, 134 out of 183 (73.2%) cheated.

The three groups differed significantly in age, Welch's *F*(2, 144.29) = 4.80, *p *=* *.010, est. ω^2^ = 0.21. A Games‐Howell post hoc test revealed that self‐initiated cheaters were significantly younger than fair players (*p *=* *.018, *d *=* *0.45). Both groups did not differ significantly in age from instructed cheaters (*p *=* *.147, *d *=* *0.23 and *p *=* *.591, *d *=* *0.14, respectively). Note that data of five participants were missing from this analysis. As the absolute age difference is minute and the potential impact within this range is very limited, age was not included as a covariate in the reported analyses.

### 2.2 Material

#### 2.2.1 HEXACO

Personality traits were assessed with the 60‐item Dutch version of the HEXACO Personality Inventory Revised (Ashton & Lee, 2009). This inventory measures the six major dimensions of personality: Honesty‐Humility, Emotionality, Extraversion, Agreeableness, Conscientiousness, and Openness to experience. Participants had to indicate to what extent they agreed or disagreed with each statement on a 5‐point Likert scale ranging from 1 (*Completely disagree*) to 5 (*Completely agree*). Each scale showed a good internal consistency using Cronbach's alpha (Cronbach, 1951): Honesty‐Humility (α = 0.71), Emotionality (α = 0.79), Extraversion (α = 0.82), Agreeableness (α = 0.77), Conscientiousness (α = 0.80), and Openness to experience (α = 0.76).

#### 2.2.2 Trivia quiz

Participants engaged in a trivia quiz with 10 open questions. Participants were not aware of the fact that the quiz was constructed in a way that it would be almost impossible to earn the bonus without looking up the correct answers to the last two questions online. The chosen eight easy questions were correctly solved by approximately 90% of the participants in a pilot study. The two questions that were each correctly solved by no more than 6% of the pilot's participants were classified as cheating‐evoking.^2^ Therefore, the probability of answering all questions correctly without cheating would be statistically highly unlikely. As a result, participants claiming the trivia bonus for answering all 10 questions correctly, including the two cheating‐evoking questions (‘Who coined the term *dinosaur*?’ as well as ‘Who wrote the autobiographical book *Prairie Tale: A Memoir*?’ as a replacement for ‘Who wrote the autobiographical book *Wishful Drinking*?’), were assumed to be cheaters. All items were pretested to ensure that the correct answers could be found within the first three search results on Google (see Domnich et al., 2015).

#### 2.2.3 CIT

The Concealed Information Test (CIT) is a method designed to measure concealed knowledge using RTs. The objective of the CIT is to verify whether the suspect possesses crime‐related information that only the perpetrator would be aware of. The method requires that the examiner identifies a number of established facts from the investigation which only the true culprit will be able to recognize. Then, the examiner presents various crime‐related details (*probes*) embedded in a set of foil items (*irrelevants*) while measuring the suspects’ behavioral responses. By choosing irrelevant items carefully, in a way that all options would seem equally plausible to unknowledgeable individuals, all items should trigger a similar response pattern. By denying the crime‐relevant items (i.e., responding “no” to probes instead truthfully admitting recognition), guilty suspects actively conceal information.

Whereas the trivia quiz manipulated the conscious act of cheating for an incentive versus fair playing, during the CIT all participants were explicitly instructed to conceal their knowledge of the correct items. Participants are required to deny knowledge both for trials containing critical items (i.e., respond “no”, hence lying) as well as irrelevant items (i.e., respond “no”, hence telling the truth). Lastly, *target* items are added to ensure that examinees pay attention to all items. These items have to be answered “yes” and are learned just before commencing the CIT.

#### 2.2.4 Follow‐up questionnaire

Motivational states were reported in a questionnaire involving five questions that participants had to rate on a 5‐point Likert scale. This questionnaire measured how well participants were able to focus on the screen during the CIT, how involved they were in the study, how well their memory was for the items of the trivia quiz and the learned target items, and how much they tried to avoid detection and appear innocent on the CIT.

#### 2.2.5 Recall and recognition

Memory for the items of the trivia quiz was assessed with a free recall followed by a recognition test. Participants first had to recall the correct answer to the two easy as well as the two cheating‐evoking questions from the trivia quiz that were subsequently used in the CIT. Afterwards, participants had to pick the correct option when presented with the probe and the four irrelevant options.

For recall, answers to the questions were each coded as either correct (1) or incorrect (0), leading to a score between 0–2 per item type (i.e., easy and cheating‐evoking questions). Using arbitrary criteria, answers were coded correct if participants recalled both the first and last name correctly (e.g., Carrie Fisher as the author of the novel *Wishful Drinking*) or when participants only recalled the last name correctly (e.g., Fisher). When an incorrect first name was entered in combination with a correct last name, or if only the first name was mentioned, the recall was coded as incorrect. For recognition, items were scored as either correct (1) or incorrect (0), leading to a score between 0 and 2 per item type (i.e., easy and cheating‐evoking questions).

### 2.3 Procedure

Participants signed up for a study concerning the relationship between mood and its impact on task performance, since announcing the true purpose of the study could distort the goal to investigate self‐initiated deceptive behavior. Participants completed the study online at their own time and on their own computer with keyboard. Approximate total participation time was 60 min.

The first part of the experiment was hosted on the survey website Qualtrics. After reading the information brochure and signing the informed consent, participants started out with the 60‐item Dutch version of the HEXACO Personality Inventory Revised (De Vries, Lee, & Ashton, 2008). To control for serious participation, three attentiveness checks were inserted (e.g., this is a control question, please indicate ‘I completely agree’). Moreover, participants could not continue to the next page within 30 s to ensure proper inspection of the material.

Then participants engaged in two seemingly relevant problem solving tasks inserted to disguise the true purpose of the study. Participants were told that they must complete these tasks alone within the timeframe of 5 min each. This was followed by a trivia quiz with 10 open (eight easy and two difficult, cheating evoking) questions. It was again made clear to the participants that they should complete the tasks alone within the timeframe of 10 min, thereby indirectly communicating that it was not allowed to use Google. Moreover, an incentive in course credits was promised when all 10 questions would be answered correctly.

Various tactics were inserted to increase the chance of cheating. To ensure a sense of privacy and anonymity, demographics were not inquired until after the experiment. Since a clear and fast reward has been shown to increase dishonest behavior (Gino & Pierce, 2009), an incentive was offered to participants who answered all trivia questions correctly. Also, since the odds of cheating are higher at the end of a series than earlier, the cheating‐evoking questions were presented as the last two questions of the quiz. The temptation to cheat is maximized when people are faced with the final opportunity to gain a reward (Effron, Bryan, & Murnighan, 2015). Lastly, participants could not continue to the next assignment within the 10‐min time frame. Pilot studies showed that this is enough time to finish the first eight easy questions (roughly two min), while leaving ample time to retrieve the correct answers online. Moreover, by setting the page timer to 10 min, sufficient time was created for participants to take an active decision of whether to cheat or not. Perhaps this also led to some frustration, thereby possibly providing entitlement and self‐justification for cheating behavior (see also Mazar, Amir, & Ariely, 2008).

For pilot purposes, participants were asked to report as specifically as possible how they knew the answers to four randomly chosen questions from the trivia quiz. In reality, these were always the same two easy and two cheating‐evoking questions that were later used in the CIT. This gave participants the opportunity to either confess to cheating, deny their cheating, or repeat that they did not know the answer. Participants also completed the 20‐item Dutch version of the Positive and Negative Affect Schedule (PANAS; Watson, Clark, & Tellegen, 1988) following the HEXACO, the quiz, and the opportunity to confess. Besides serving for pilot purposes, this additional mood questionnaire facilitated the cover story, concealing the true purpose of the study.

Then, participants continued to the second part of the experiment, starting with the memory detection test, programmed in Inquisit 4.0 by Millisecond Software. Participants were told to hide their knowledge of the correct answers in the trivia quiz they completed previously. Upon successful concealment,^3^ an additional incentive in course credits would be awarded. The CIT included answers to two of the easy questions as to measure a baseline‐response to known items, as well as the answers to the two cheating‐evoking questions that participants might have cheated on. Per question, the CIT included the correct answer, four incorrect answers serving as irrelevant options, and a target item. Hence, if Rome was the probe stimulus, the target was Madrid, and the irrelevant stimuli were Paris, Vienna, London, and Berlin.

Participants had to respond to the question “*Is this the correct answer?”* by pressing either the left (A‐key) button for YES or the right (L‐key) button for NO on their keyboard. The question and the response keys remained on the screen during the entire test as a reminder. Participants were instructed to respond with YES only to the target items and NO to all other stimuli (i.e., both the correct answers of the trivia quiz and the irrelevant options). Each trial consisted of one answer (e.g., Berlin) being displayed as a word in the middle of the screen for exactly 1,500 ms. If the participant did not give a response within the maximum response deadline of 800 ms, the message TOO SLOW appeared in red color above the stimulus for 200 ms. If the participant's response was incorrect, that is responding with NO for target items or with YES to probe or irrelevant items, the word WRONG appeared in red color below the stimulus for 200 ms. Response latency was measured from the onset of the stimulus on the screen until one of the response keys was pressed. After key‐press or after the 1,500 ms presentation time, the next stimulus appeared on the screen with an inter‐stimulus interval (ISI) of either 250, 500, or 750 ms to prevent response preparation.

In order to ensure proper understanding of the task and instructions, each participant had to pass through a stepwise practice procedure that allowed participants to become used to the speed and requirements of the task. Each of the three practice phases of the memory detection test consisted of 24 trials. In the first practice phase, participants could pace the speed of the trial sequence themselves, so that a new stimulus only appeared after a key press. Feedback was given upon an erroneous response, but the TOO SLOW message was not presented. Participants could proceed to the next phase when their target accuracy was at least 75%; otherwise the first practice phase was repeated until this requirement was met. In the second phase the 1,500 ms stimulus presentation was added, so that the next trial would automatically appear upon key press or after 1,500 ms. Again, feedback was given upon an erroneous response, but the TOO SLOW message was never presented. Participants could only proceed to the next phase when their target accuracy was at least 50% and as an additional requirement, when their mean response latency was below 800 ms, otherwise this practice phase was repeated until their performance was satisfactory. The last practice phase was identical to the full test, including the WRONG and TOO SLOW feedback. Participants could proceed to the actual test only when their target accuracy was at least 50% and when their mean response latency was below 800 ms.

In the actual CIT, all words were presented in a 1:1:4 ratio; that is, of the total 480 trials in the test, 80 were probe stimuli, 80 were target stimuli, and 320 were irrelevant stimuli, so that each stimulus was displayed exactly 20 times. The sequence of the stimuli was randomized, as well as the question types (i.e., easy and cheating‐evoking) and the ISI.

After completing the CIT, participants were presented with a questionnaire designed to assess their attention to the tasks, involvement in the experiment, memory for the stimuli, and their motivation to avoid detection in the CIT on a 5‐point Likert scale (ranging from *1* = *not at all* to *5* = *very much so*). Then, participants were told that the experiment was finished and they did not have to hide any information anymore. They had to give honest answers to the four CIT questions (i.e., the two easy questions as well as the two cheating‐evoking questions) in a free‐recall and subsequently in a multiple‐choice recognition test. Only thereafter demographic information was asked, including gender, age, and field of study. Lastly, participants were debriefed and compensated for participation.

## 3 Results

### 3.1 CIT

Trials to which no response was recorded (i.e., RTs larger than 1,500 ms) were excluded from all subsequent analyses. Moreover, trials with an incorrect response (i.e., pressing NO for target items and/or YES for probe and irrelevant items) as well as trials with a RT below 150 ms and above 800 ms (see also Verschuere, Crombez, Degrootte, & Rosseel, 2010) were excluded from response latency analysis. Of all 294 participants who completed the CIT, three participants did not give the correct answer to one of the easy questions, and CIT trials addressing those questions were excluded from analysis. CIT trials addressing the cheating‐evoking questions for fair players who admitted to have searched for the answer online without filling it in on the trivia quiz were excluded from the analysis. On average, 445 trials (92.7%) per participant were included in the analyses (range: 61.5%–98%). All analyses used an alpha level of 0.05. Effect sizes for the anova are reported using Cohen's *f*. For follow‐up contrasts Cohen's *d* is used.^4^ Cohen's *d* for within‐subject and between‐subject comparisons is annotated as *d*_within_ and *d*_between_. As a rule of thumb, Cohen (1988) proposed 0.20, 0.50 and 0.80 as thresholds for “small,” “moderate,” and “large” effects, respectively, for *d* values and 0.10, 0.25, and 0.40 as thresholds for “small,” “moderate,” and “large” effects, respectively, for *f* values. Moreover, JZS Bayes factors were computed, using JASP software version 0.8.4, which are numerical values quantifying the odds ratio between the null and the alternative hypothesis given the data. A default JZS prior with scaling factor *r *=* *0.707 was used for the alternative hypothesis (see Rouder, Speckman, Sun, Morey, & Iverson, 2009). The JZS Bayes factors are reported as either in favor of the null or the alternative hypothesis. Using Jeffreys’s (1961) criteria, a Bayes factor of three or more is taken as substantial evidence for the respective hypothesis. Lastly, the area under the Receiver Operating Characteristic (ROC) curve was calculated. This statistic describes the detection efficiency of the CIT in differentiating between knowledgeable and unknowledgeable individuals, computed across all possible cut‐off points on the detection score. The ROC area varies between 0 and 1, with a chance level of 0.5 (for a more detailed description, see Lieblich, Kugelmass, & Ben‐Shakhar, 1970).

#### 3.1.1 RTs

In the main analysis, a 3 (Condition: self‐initiated cheater vs. instructed cheater vs. fair player, between‐participants) by 2 (Stimulus: probe vs. irrelevant, within‐participants) by 2 (Question: easy vs. cheating, within‐participants) mixed anova was conducted on reaction times in milliseconds. Although RTs were the prime outcome measure as they are typically more valid than error rates (Kleinberg & Verschuere, 2015), the same anova was conducted and reported for error rates.

The mixed anova revealed a significant main effects of Stimulus, *F*(1, 288) = 188.59, *p *<* *.001, *f* = 0.81 (i.e., longer RTs to probes than to irrelevants), and Question, *F*(1, 288)  = 136.48, *p *<* *.001, *f *=* *0.69 (i.e., longer RTs to the cheating‐evoking questions than to the easy questions). Significant interactions were revealed between Condition and Stimulus, *F*(2, 288) = 5.48, *p *=* *.005, *f *=* *0.20 (i.e., larger probe‐irrelevant difference for self‐initiated and instructed cheaters than for fair players), and between Condition and Question, *F*(2, 288) = 3.33, *p *=* *.037, *f *=* *0.15 (i.e., greater difference in RTs between the cheating‐evoking and easy questions for self‐initiated and instructed cheaters compared to fair players). These effects are collapsed under a statistically significant three‐way interaction of Condition by Stimulus by Question, *F*(2, 288) = 7.38, *p *=* *.001, *f *=* *0.23. Table 1 shows the mean RTs for each cell of the design.

**Table 1:** Mean reaction times (in ms) and mean error rates (in %; *SD*s in parentheses) for easy and difficult questions for self‐initiated cheaters, instructed cheaters, and fair players

Self‐Initiated Cheaters (*n *=* *142)					Instructed Cheaters (*n *=* *107)				Fair Players (*n *=* *45)			
	Probe	Irrelevant	*p*‐value	*d*_within_	Probe	Irrelevant	*p*‐value	*d*_within_	Probe	Irrelevant	*p*‐value	*d*_within_
RTs												
Easy questions	468 (43)	452(36)	*p *<* *.001	0.65 [0.50; 0.81]	470 (40)	455 (31)	*p *<* *.001	0.59 [0.42; 0.77]	478 (41)	461 (29)	*p *<* *.001	0.59 [0.23; 0.96]
Difficult questions	493 (49)	472 (39)	*p *<* *.001	0.81 [0.63; 0.99]	499 (41)	475 (34)	*p *<* *.001	0.98 [0.76; 1.21]	483 (34)	481 (33)	*p *=* *.607	0.10 [−0.28; 0.47]
Error rates												
Easy questions	5.46 (5.91)	1.95 (1.77)	*p *<* *.001	0.60 [0.45; 0.76]	5.86 (8.16)	2.61 (4.75)	*p *<* *.001	0.55 [0.37; 0.72]	7.67 (7.45)	2.39 (3.37)	*p *<* *.001	0.75 [0.38; 1.12]
Difficult questions	5.83 (6.85)	1.92 (1.85)	*p *<* *.001	0.58 [0.44; 0.73]	6.33 (7.73)	2.40 (4.74)	*p *<* *.001	0.67 [0.48; 0.85]	3.44 (6.58)	2.88 (3.51)	*p *=* *.479	0.11 [−0.24; 0.45]

To narrow down the three‐way interaction, a 3 (Condition: self‐initiated cheater vs. instructed cheater vs. fair player, between‐participants) by 2 (Stimulus: probe vs. irrelevant, within‐participants) mixed anova was conducted for the easy and the cheating‐evoking questions separately.

For the easy questions, the anova revealed a significant main effect of Stimulus, *F*(1, 291) = 94.18, *p *<* *.001, *f *=* *0.57, and no significant interaction between Condition and Stimulus *F*(2, 291) = 0.14, *p *=* *.867, *f *=* *0.03. This indicates that there was a clear CIT‐effect across the conditions and since all participants knew the correct answer, no differences emerged between the three conditions.

For the cheating‐evoking questions, the anova revealed a significant main effect of Stimulus, *F*(1, 288) = 89.31, *p *<* *.001, *f *=* *0.56, and a significant interaction between Condition and Stimulus *F*(1, 288) = 13.33, *p *<* *.001, *f *=* *0.30. This indicates that the CIT‐effect differed according to the condition. Planned contrasts in a follow‐up one‐way anova on RT difference scores (i.e., RT_probe_ – RT_irrelevant_) revealed a significant difference between the two conditions in which participants knew the correct answer to the cheating‐evoking questions (i.e., self‐initiated and instructed cheaters) versus the fair players, who did not know the correct answers, *t*(288) = 5.08, *p* < .001, *d*_between_ = 0.83, BF = 19,512.42 in favor of the alternative hypothesis. There was no significant difference in the RTs between the self‐initiated and instructed cheaters, *t*(288) = −1.20, *p *=* *.232, *d*_between_ = 0.15, BF = 3.62 in favor of the null hypothesis.

#### 3.1.2 ROC

In order to analyze detection efficiency of classifying individuals as knowledgeable (i.e., cheaters) versus unknowledgeable (i.e., fair players), we compared the distribution of the within‐person detection scores for both self‐initiated cheaters and instructed cheaters with the detection score distribution of fair players. For both of these comparisons, we computed the ROC area as well as their respective 95% confidence intervals. Analysis reveals that detection efficiency was significantly larger than a chance area of 0.50 for both self‐initiated and instructed cheaters with ROC areas of 0.73 [0.64; 0.81] and 0.78 [0.70; 0.86], respectively.

#### 3.1.3 Error rates

The 3 (Condition: self‐initiated cheater vs. instructed cheater vs. fair player, between‐participants) by 2 (Stimulus: probe vs. irrelevant, within‐participants) by 2 (Question: easy vs. cheating, within‐participants) mixed anova on error rates revealed a significant main effect of Stimulus, *F*(1, 291) = 148.99, *p *<* *.001, *f *=* *0.72 (i.e., more errors to probes than to irrelevants), that was qualified by the Condition × Question, *F*(2, 291) = 3.59, *p *=* *.029, *f *=* *0.16, and Stimulus × Question, *F*(1, 291) = 4.39, *p *=* *.037, *f *=* *0.12 interactions. These effects are collapsed under the three‐way significant interaction of Condition × Stimulus × Question, *F*(2, 291) = 6.71, *p *=* *.001, *f *=* *0.21. Table 1 shows the mean errors for each cell of the design.

To narrow down the three‐way interaction, a 3 (Condition: self‐initiated cheater vs. instructed cheater vs. fair player, between‐participants) by 2 (Stimulus: probe vs. irrelevant, within‐participants) mixed anova was conducted for the easy and the cheating‐evoking questions separately.

For the easy questions, the anova revealed a significant main effect of Stimulus, *F*(1, 291) = 102.25, *p *<* *.001, *f *=* *0.59, and no significant interaction between Condition and Stimulus *F*(2, 291) = 1.88, *p *=* *.155, *f *=* *0.11. This indicates that there was a clear CIT‐effect across the conditions and since all participants knew the correct answer, no differences emerged between the three conditions.

For the cheating‐evoking questions, the anova revealed a significant main effect of Stimulus, *F*(1, 291) = 47.44, *p *<* *.001, *f *=* *0.40, and a significant interaction between Condition and Stimulus *F*(2, 291) = 5.55, *p *=* *.004, *f *=* *0.20. Planned contrasts in a follow‐up one‐way anova conducted on the error rate difference scores (i.e., error rate_probe_ – error rate_irrelevant_) revealed a significant difference between the two conditions in which participants knew the correct answer to the cheating‐evoking questions (i.e., self‐initiated and instructed cheaters) versus the fair players, who did not know the correct answers, *t*(291) = 3.33, *p* = .001, *d*_between_ = 0.54, *BF* = 27.32 in favor of the alternative hypothesis. There was no significant difference in the error rates difference scores between self‐initiated and instructed cheaters, *t*(291) = −0.04, *p *=* *.972, *d*_between_ = 0.00, BF = 7.13 in favor of the null hypothesis.

### 3.2 Hexaco

A one‐way anova was conducted on the data of the 366 participants who successfully completed the HEXACO personality measure, to determine whether reported Honesty‐Humility differed between the three experimental conditions. Analysis revealed no statistically significant difference between the three conditions on Honesty‐Humility, *F*(2) = 1.95, *p *=* *.144, *f *=* *0.11.

For completion sake, we contrasted the groups on Honesty‐Humility. The planned contrast did not show a statistically significant difference between self‐initiated cheaters (*M *=* *3.18, *SD* = 0.54) and fair players (*M *=* *3.30, *SD* = 0.56, *t*(363) = −1.51, *p *=* *.133, *d*_between_ = 0.22, BF = 2.15 in favor of the null hypothesis). Post hoc comparisons with Bonferroni correction revealed that self‐initiated cheaters as well as fair players did not differ from the control group (i.e., instructed cheaters; *M *=* *3.14, *SD* = 0.53, *p *=* *1.00, *d*_between_ = 0.07, and *p *=* *.149, *d*_between_ = 0.30, respectively).

Also for sake of completion, additional anovas showed no significant effects for the remaining personality factors, Emotionality, *F*(2) = 0.13, *p *=* *.881, *f *=* *0.03, Agreeableness, *F*(2) = 0.28, *p *=* *.760, *f *=* *0.04, Conscientiousness, *F*(2) = 0.38, *p *=* *.684, *f *=* *0.04, or Openness to Experience, *F*(2) = 1.52, *p *=* *.221, *f *=* *0.09. There was a statistically significant difference between the three conditions on Extraversion, Welch's *F* (2, 147.10) = 3.59, *p *=* *.030, est. ω^2^ = 0.01. A Games‐Howell post hoc test that contrasted self‐initiated cheaters (*M *=* *3.62, *SD* = 0.54) with fair players (*M *=* *3.37, *SD* = 0.78) revealed a marginally significant effect of Extraversion (*p *=* *.053, *d*_between_ = 0.41). No significant differences in levels of Extraversion emerged between the self‐initiated cheaters and fair players with the control group of instructed cheaters (*M *=* *3.51, *SD* = 0.54, *p *=* *.169, *d*_between_ = 0.20 and *p *=* *.402, *d*_between_ = 0.22, respectively). Table 2 shows the descriptive statistics and internal consistency.

**Table 2:** Mean scores (*SD*s in Parentheses) on the factors of 60‐item Dutch version of the HEXACO‐PI‐R on a 5‐point Likert scale

	Self‐Initiated Cheaters (*n *=* *175)	Fair Players (*n *=* *61)	Instructed Cheaters (*n *=* *130)	*p*‐value	Effect Size	Internal Consistency
Honesty‐humility	3.18 (0.54)	3.30 (0.56)	3.14 (0.53)	*p *=* *.144	*f *=* *0.11	α = 0.71
Emotionality	3.24 (0.62)	3.29 (0.52)	3.26 (0.66)	*p *=* *.881	*f *=* *0.03	α = 0.79
Extraversion	3.62 (0.54)	3.37 (0.78)	3.51 (0.54)	*p *=* *.030	ω^2^ = 0.01	α = 0.82
Agreeableness	3.13 (0.58)	3.10 (0.60)	3.08 (0.59)	*p *=* *.760	*f *=* *0.04	α = 0.77
Conscientiousness	3.47 (0.65)	3.39 (0.62)	3.47 (0.61)	*p *=* *.684	*f *=* *0.04	α = 0.80
Openness to experience	3.33 (0.61)	3.49 (0.63)	3.41 (0.66)	*p *=* *.221	*f *=* *0.09	α = 0.76

### 3.3 Follow‐up questionnaire

Of all 294 participants who successfully completed the CIT, one participant did not complete the follow‐up questionnaire and was therefore excluded from this analysis. Table 3 shows the mean scores for each cell of the design.

**Table 3:** Mean scores (*SD*s in parentheses) on the follow‐up questionnaire on a 5‐point Likert scale

	Self‐Initiated Cheaters (*n *=* *141)	Fair Players (*n *=* *45)	Instructed Cheaters (*n *=* *107)	*p*‐value	Effect Size
Reported focus on the computer screen	3.48 (1.17)	3.58 (1.27)	3.66 (1.17)	*p *=* *.462	*f *=* *0.07
Reported involvement in the experiment	4.04 (0.89)	4.00 (1.00)	4.04 (0.91)	*p *=* *.970	*f *=* *0.00
Reported memory for the answers of the quiz	3.96 (0.89)	3.56 (1.01)	3.97 (0.81)	*p *=* *.043	ω^2^ = 0.02
Reported memory for the target items	4.09 (0.85)	4.16 (0.90)	4.15 (0.87)	*p *=* *.806	*f *=* *0.03
Reported effort to hide knowledge	4.42 (0.71)	4.29 (1.01)	4.32 (0.90)	*p *=* *.649	*f *=* *0.06

The three conditions did not differ in their reported focus, *F*(2) = 0.77, *p *=* *.462, *f *=* *0.07, involvement, *F*(2) = 0.03, *p *=* *.970, *f *=* *0.00, memory for the target items, *F*(2) = 0.22, *p* = .806, *f *=* *0.03, or motivation to avoid detection, *F*(2) = 0.649, *p *=* *.523, *f *=* *0.06. Analysis revealed a statistically significant difference between the three conditions on their reported memory for the answers of the trivia quiz, *W*elch's *F*(2, 115.59) = 3.49, *p *=* *.043, est. ω^2^ = 0.02.

A Games‐Howell post hoc test showed that the fair players (*M *=* *3.56, *SD* = 1.01) differed significantly from both instructed (*M *=* *3.97, *SD* = 0.81, *p *=* *.044, *d*_between_ = 0.47) and self‐initiated cheaters (*M *=* *3.96, *SD* = 0.89, *p *=* *.051, *d*_between_ = 0.43) on reported memory for the answers of the quiz. The latter two conditions did not differ significantly (*p *=* *.990, *d*_between_ = 0.01).

### 3.4 Recall and recognition

Of all 294 participants who successfully completed the CIT, one participant did not complete the free recall and recognition tasks that assessed memory for the four critical items of the trivia quiz.

#### 3.4.1 Recall

A one‐way anova for the easy questions revealed a clear ceiling effect for self‐initiated cheaters (*M *=* *1.99, *SD* = 0.08), instructed cheaters (*M *=* *1.96, *SD* = 0.23), and fair players (*M *=* *1.89, *SD* = 0.38), with no significant difference in recall rate between the conditions, *Welch's F* (2, 88.71) = 2.35, *p *=* *.101, est. ω^2^ = 0.01. A second one‐way anova for the cheating‐evoking questions revealed a significant difference between self‐initiated cheaters (*M *=* *1.64, *SD* = 0.69), instructed cheaters (*M* = 1.55, *SD* = 0.73), and fair players (*M *=* *0.16, *SD* = 0.47), Welch's *F* (2, 142.53) = 149.11, *p *<* *.001, est. ω^2^ = 0.50. A Games‐Howell post hoc test showed that the fair players scored significantly lower than both instructed (*p *<* *.001, *d*_between_ = 2.08) and self‐initiated cheaters (*p *<* *.001, *d*_between_ = 2.29). The latter two conditions did not differ significantly (*p* = .609, *d*_between_ = 0.13).

#### 3.4.2 Recognition

A one‐way anova for the easy questions revealed a ceiling effect of recognition for self‐initiated cheaters (*M *=* *1.99, *SD* = 0.08), instructed cheaters (*M *=* *1.98, *SD* = 0.14), and fair players (*M *=* *1.98, *SD* = 0.15), with no significant difference in recognition rate between the conditions, *F*(2)  = 0.45, *p *=* *.641, *f *=* *0.05. A second one‐way anova for the cheating‐evoking questions revealed a significant difference between self‐initiated cheaters (*M *=* *1.98, *SD* = 0.14), instructed cheaters (*M *=* *1.98, *SD* = 0.14) and fair players (*M *=* *0.78, *SD* = 0.70), Welch's *F* (2, 99.82) = 64.80, *p *<* *.001, est. ω^2^ = 0.30. A Games‐Howell post hoc test showed that the fair players scored significantly lower than both instructed (*p *<* *.001, *d*_between_ = 3.01) and self‐initiated cheaters (*p *<* *.001, *d*_between_ = 3.29). The latter two conditions did not differ significantly (*p* = .989, *d*_between_ = 0.00).

## 4 Discussion

To examine the external validity of detection of deception research, the current study explored whether the cognitive signature of lying differs for instructed versus self‐initiated cheating. Using a well‐established paradigm (CIT; Lykken, 1959; Verschuere, Ben‐Shakhar, & Meijer, 2011) and measure (RTs; Suchotzki et al., 2017), we instructed some participants to cheat on a trivia quiz, while providing others with an opportunity and incentive to cheat without an explicit instruction. Results of 294 memory detection tests replicated the typically observed response pattern (i.e., increased response latency for relevant items), with no differences between instructed and self‐initiated cheaters. This result indicates that the cognitive signature of lying extends to self‐initiated cheating, thereby strengthening the external validity of CIT studies using RTs. Additionally, the CIT adequately discriminated between cheating participants and fair players.

### 4.1 The cognitive signature of lying

From a cognitive perspective, lying is regarded as a cognitive process that imposes a greater burden on our executive functions than truth telling, which is viewed as the default in human communication. A growing literature, using a diverse array of paradigms and measures, provides increasing support for the idea that lying is typically more demanding than truth telling (Caso et al., 2005; Debey, Ridderinkhof, De Houwer, De Schryver, & Verschuere, 2015; Debey, Verschuere, & Crombez, 2012; Duran et al., 2010; Vrij et al., 1996; Walczyk et al., 2003). In the current paradigm this response conflict is reflected by the prolonged RT when deliberately denying knowledge compared to responding to neutral irrelevant options. Using this contrast between probes and irrelevant items, the CIT distinguished cheaters from fair players (*d* = 0.83).

While various approaches using the cognitive load theory have emerged, the specific underlying mechanisms that generate the difference between liars and truth‐tellers remains debated. Researchers have summoned to investigate how various executive functions such as working memory and response inhibition exactly contribute to this cognitive signature of lying (see Blandón‐Gitlin, Fenn, Masip, & Yoo, 2014; Gombos, 2006) and whether boundary conditions apply. By investigating the influence of self‐initiated (as opposed to laboratory‐induced) behavior, the current findings shed a light on the role of intent on the cognitive processes underlying deception.

Recent insights suggest that Response Inhibition (RI; Verschuere, Crombez, Koster, Van Bockstaele, & De Clercq, 2007) plays a critical role in obtaining the cognitive signature of lying, in particular for the RT‐CIT (Suchotzki et al., 2017). The RT‐CIT has been found to be influenced by neither item saliency (Klein Selle et al., 2017) nor by increased motivation to avoid detection (Kleinberg & Verschuere, 2016). This could explain why no differences in detection accuracy were found between the participants who were instructed to cheat on the quiz (mimicking the typical laboratory set‐up) and the participants who cheated on the quiz on their own initiative (*d *=* *0.15, as well as substantial evidence for the null hypothesis; BF = 3.62 in favor of the null hypothesis).

The data corroborate the hypothesis of the cognitive impact on lying, with the addition that it is not restricted to explicitly instructed cheating, but can also be observed for intentional, self‐initiated cheating.

### 4.2 Who cheats?

Literature in clinical psychology has focused on personality traits that causes some individuals to cheat with a higher likelihood than others. For instance, psychopathy (Nathanson, Paulhus, & Williams, 2006) has been linked to increased likelihood of cheating. In fact, individual propensity to cheat has been deemed so important that Honesty‐Humility was added as an additional factor to the Big Five in the HEXACO model of personality (Hilbig & Zettler, 2015). Social psychology, on the other hand, has focused on state factors that prompt individuals to cheat, depending on the situational context. For example, cheating behavior increased when participants were placed in a dark room (Zhong, Bohns, & Gino, 2010), were given limited time to think (Shalvi, Eldar, & Bereby‐Meyer, 2012), when monetary rewards were given to charity (Lewis et al., 2012), or when others could benefit from their cheating behavior (Gino, Ayal, & Ariely, 2013).

While participants made the active decision to cheat or not, no significant differences emerged in Honesty‐Humility between the self‐initiated cheaters and fair players. The high cheating rate (75%) that was found in the current study, therefore, seems to be influenced by the situation, rather than a reflection of differences in personality traits. Since we created a fairly unambiguous and strong manipulation (i.e., an untraceable opportunity to cheat for a reward in an online setting), room for individual differences was limited. Hence, the obtained results are relatively uniform across participants. This concept of a *strong situation* (Ickes, 1982; Lissek, Pine, & Grillon, 2006; Mischel, 1977; Monson & Snyder, 1977), as described in social psychology, might be an important limitation to measuring interpersonal differences in the current experimental setting. A less anonymous situation in which participants are more hesitant to transgress experimental rules might reveal individual differences that did not emerge in the current study.

### 4.3 Applied implications

The current study speaks to the ecological validity of the CIT. While the CIT has been tested in hundreds of studies, those studies typically used artificial settings, with trivial stimuli (e.g., play cards or mock crime items), undergraduate participants, little or no incentives, and an explicit instruction to cheat or lie. More recently, researchers have started to manipulate those factors exploring whether and how they influence CIT validity. Several factors have been found to have little or no affect, including stress and arousal during crime and test (klein Selle et al., 2017; Peth, Vossel, & Gamer, 2012). Other factors, such as time between encoding and test (Carmel, Dayan, Naveh, Raveh, & Ben‐Shakhar, 2003; Nahari & Ben‐Shakhar, 2011; Peth et al., 2012) have found to affect CIT validity. The current study adds to this growing body of research by indicating that self‐initiated cheaters were indistinguishable in their responses from instructed cheaters.

Besides tackling the effect of self‐initiated versus instructed cheating, the current paradigm also allows for testing incidentally acquired, real‐world memories (see also Meixner & Rosenfeld, 2014) as opposed to controlled, artificial encoding of stimuli. That is, in most laboratory studies participants typically acquire information through mock crime procedures in which a certain object has to be stolen from a specified location. This results in an artificial focus on these stimuli, which have to be memorized for the sake of the experiment. In the current paradigm, participants acquired the critical information in a natural setting. By using various distracting filler tasks and a plausible cover story, it can be assumed that participants did not deliberately retain the information until the memory detection test. While this new method was particularly designed for external validity purposes rather than demonstrating the CIT‐effect, moderate to large effect sizes were found. These results are promising and should encourage further field application of memory detection.

### 4.4 Limitations and suggestions for future research

This study is not without its limitations. First, the goal of the current paradigm was to compare self‐initiated cheating with instructed cheating and fair playing, so it was crucial to elicit spontaneous cheating on the trivia quiz. It bears mentioning that some participants might have known the answer to the cheating‐evoking questions and actually did not cheat. While the anonymous online setting cannot confirm cheating with absolute certainty, pilot studies in similar samples showed that the chance of knowing the answers to both questions was statistically very small. Still, in a subsequent experiment, this could be prevented by monitoring the participants’ screen or asking non‐existing questions of which the answers can only be found on a webpage designed by the experimenter.

Second, a question can be raised as to whether participants considered looking up the correct answers online as cheating, when the instructions merely required participants to solve the quiz “alone.” Yet this wording was specifically chosen to create a situation in which participants could spontaneously initiate cheating behavior. Explicit instructions *not* to use Google were avoided, since it could have provoked the opposite effect, besides possibly revealing the true nature of the study. As a drawback of these instructions, participants might have assumed it was not necessarily prohibited. Interestingly, a substantial number of self‐initiated cheaters (40%) felt the need to justify their correct response on the trivia quiz, when presented with an opportunity to confess. This might be an indication that using Google was in fact considered rule‐breaking behavior.

Third, the study was run online. Participants used their own computer and Internet connection throughout the various stages of the 60‐min experiment. While attentiveness during online testing has been voiced as a concern, various researchers have positively evaluated the use of online platforms for psychological studies (see Bartneck, Duenser, Moltchanova, & Zawieska, 2015; Mason & Suri, 2012). Despite fully acknowledging the lack of complete control, it is noteworthy that (1) attentiveness checks were inserted in the questionnaires and that very few participants failed the test; (2) incentives were included for good performance; (3) participants had extensive practice before commencing the CIT; and (4) strict exclusion criteria were used. Given the low number of participants who were excluded based on inattentiveness in the HEXACO questionnaire or in the CIT, as well as the high trial accuracy in the CIT for the remaining participants, we think that memory detection could be meaningfully investigated in the current online setting.

## 5 Conclusions

The results imply that the cognitive signature of lying (slower responses when lying as compared to truth telling) extends to self‐initiated cheating. This finding is encouraging from an ecological validity perspective and may pave the way for further field implementation of memory detection.

## Notes

1 This criterion ensured that only those participants who understood the instructions and took the task seriously were included in the results (see Materials).

2 Pilot questionnaires revealed that the eight easy questions were each correctly answered by 88–100% of the sample, whereas the cheating‐evoking questions were correctly answered by 0% (Who coined the term dinosaur?), 3% (Who wrote the autobiographical book Wishful Drinking?), and 6% (Who wrote the autobiographical book Prairie Tale: A Memoir?). Thus, the likelihood of answering two of these questions correctly is close to 0. When answering both cheating‐evoking questions correctly, participants were labeled “cheaters.”

3 Participants were given an additional bonus when their individual CIT score (*d*_CIT_ = (M_RT(probes)_ – M_RT(irrelevants)_)/*SD*_RT(irrelevants)_) was below 0.2 (see also Noordraven & Verschuere, 2013).

4 The probe‐irrelevant within‐subject contrast was calculated as *d*_within_ = *M*(RT_(probes)_ *– *RT_(irrelevants)_ */√*(*SD*_(probes)_^*2*^ *+ SD*_(irrelevants)_^2^ – 2**r*SD*_(probes)_ **SD*_(irrelevants)_ *,* where *r* is the Pearson correlation between *RT*_(probes)_ and *RT*_(irrelevants)_. The between‐subject contrast was calculated as *d*_between_ = (*M*_RT(probe‐irrelevant difference group 1)_ – *M*_RT(probe‐irrelevant difference group 2)_/*√(((n*_(group 1_ – 1)**SD*_(probe‐irrelevant difference group 1)_^2^ + (*n*_(group 2_ – 1)**SD*_(probe‐irrelevant difference group 2)_^2^)/*n*_group1_ + *n*_group2_ – 2), see also Lakens (2013) and Suchotzki et al. (2017).
